# Efficacy of Female Rat Models in Translational Cardiovascular Aging Research

**DOI:** 10.1155/2014/153127

**Published:** 2014-12-31

**Authors:** K. M. Rice, J. C. Fannin, C. Gillette, E. R. Blough

**Affiliations:** ^1^Center for Diagnostic Nanosystems and Robert C. Byrd Biotechnology Science Center, Marshall University, Room 241D, 1700 3rd Avenue, Huntington, WV 25755-1090, USA; ^2^Department of Pharmacology, Physiology and Toxicology, Joan C. Edwards School of Medicine, Marshall University, Huntington, WV, USA; ^3^Department of Pharmacy Practice, Administration, and Research, School of Pharmacy, Marshall University, Huntington, WV, USA; ^4^Department of Pharmaceutical Sciences and Research, School of Pharmacy, Marshall University, Huntington, WV, USA

## Abstract

Cardiovascular disease is the leading cause of death in women in the United States. Aging is a primary risk factor for the development of cardiovascular disease as well as cardiovascular-related morbidity and mortality. Aging is a universal process that all humans undergo; however, research in aging is limited by cost and time constraints. Therefore, most research in aging has been done in primates and rodents; however it is unknown how well the effects of aging in rat models translate into humans. To compound the complication of aging gender has also been indicated as a risk factor for various cardiovascular diseases. This review addresses the systemic pathophysiology of the cardiovascular system associated with aging and gender for aging research with regard to the applicability of rat derived data for translational application to human aging.

## 1. Introduction

In 2005, cardiovascular disease (CVD) was the underlying cause of death in 864,480 of the approximately 2.5 million total deaths in the United States [1, 2]. CVD is the leading cause of death and the most costly disease in America and is expected to increase in costs to $1.48 trillion by 2030 [3]. Although the focus has been to reduce the modifiable risk factors for cardiovascular disease (such as lipid levels, diabetes, and sedentary lifestyle), the unmodifiable risk factor, aging, is a major risk factor for coronary disease, hypertension, congestive heart failure, and stroke [4]. The number of the aged in the United States is projected to increase by more than 20% by the year 2030. This growth in the elderly population is expected to significantly test our already overloaded health care system.

Although years of research have been conducted in regard to aging, we are still a long way from understanding the intricacies of age-related changes in human physiology, in particular the cardiovascular system. Whether aging in animal models mimics many of the cardiovascular changes seen in humans is not well understood. In addition, the process of delineating the effect gender has on aging provides yet another variable to be considered.

## 2. Discussion

### 2.1. Aging and Cardiovascular Disease

Aging is believed by some to be a progressive disorder that decreases an organism's ability to maintain reproductive capacity and “normostasis” [5]. Indeed a strong correlation exists between aging and higher incidence of several diseases including dementia, Parkinson's disease, diabetes, cancer, and Alzheimer's disease [5]. In the cardiovascular system, aging is positively correlated with an increasing risk of cardiac problems including arrhythmias and is a major independent risk factor for cardiovascular-related morbidity and mortality [6]. Indeed, more than 70% of males and females over 75 years of age present some clinically evident cardiovascular disease (CVD) [7]. In addition to age, CVD risk also appears to vary with sex. CVD is the number one killer of women in Western nations [8]. Among women, more than 200,000 of the 454,613 total CVD deaths occurred in those above 85 years of age [2]. Although cardiovascular risk increases with age in both sexes, the increase in age-associated risk appears to be sharper in women [9]. Why CVD risk may differ between men and women is not well understood but may be related to differences in age-associated cardiovascular function [10]. Whereas congestive heart failure in men is oftentimes due to systolic insufficiencies, congestive heart failure in women is often related to diastolic dysfunction [10, 11]. The incidences of ischemia, cardiac failure, and cardiac rupture in addition to ventricular remodeling have also been shown to differ between genders with aging and often lead to worse outcomes in women [12–15]. Recent data suggests that premenopausal women have a decreased risk of CVD compared to men of comparable age [16, 17]. This cardioprotective benefit appears to be lost over time as the risk of developing cardiovascular disease in postmenopausal women increases to a rate that is similar to that observed in men [18]. The reason(s) for this increase in CVD risk following menopause is currently unclear; however, it is well known that early menopause is associated with an increased risk for coronary heart disease and cardiovascular disease death [19–21]. This finding is thought to be related, at least in part, to diminished estrogen exposure [22]. Consistent with this notion, each year of increasing age at natural menopause has been found to be associated with a 2% increase in total cardiovascular mortality [22, 23]. Whether differences in estrogen alone or changes in estrogen along with other factors can fully explain the sex related differences in age-related cardiovascular function is unknown and requires further investigation.

### 2.2. Aging Female Rat Models

Human aging research is limited due to cost, differences in lifestyle/history, and more importantly the time required for data collection as well as analysis of that particular system. Importantly, there are a number of different animal models that can be used to acquire information on how aging affects the female cardiovascular system. The most applicable aging female animals studied include primates. Primates are the closest in regard to female human aging due to the fact that they are the only species to undergo menstrual sloughing of the endometrial lining. Nonetheless, it is important to note that female primates do not experience menopause [25]. In addition, primate research is oftentimes ethically difficult to perform and costly.

Rats are widely used in aging research as they exhibit a relatively short lifespan and are genetically quite homogenous. Among the various strains of rats, the Wistar, F344, and F344/Brown Norway are the most common used aging female rat models [26–30]. Although rats do not experience menses, they do experience estrus cycling and ovarian aging.  Table 1 presents the stages of ovarian aging in female rats. Reproductive maturity is reached at five months when there is an estrous cycle that lasts four to five days. Aging rats exhibit periods of persistent estrous cycle which consists of elevated and constant levels of estradiol, low levels of progesterone, and lack of luteinizing hormone (LH) surges, in addition to ovulation [24, 31, 32]. Ovarian decline occurs between six and eighteen months depending on the rodent strain and is characterized by low levels of estradiol and progesterone, with little or no developing follicles and increased prolactin secretion [24, 31, 32].

Comparisons between aging research in female rats models and humans can be complicated due to the differences in the mechanisms of ovarian/hormone aging in human females and its potential impact on cardiovascular disease. Though not fully understood, it is thought that the loss of the hormones estrogen and progesterone in aging human females is due to the decrease in the ovarian follicular reserve [33]. Conversely, aging female rats experience a persistent estrous cycle due to chronic anovulation which consist of pseudopregnant/disestrus estrogen levels as well as high progesterone levels from increased ovulation and the corpora lutea [33]. Therefore, reproductive senescence in female rats consists of alterations in the hypothalamic-pituitary axis while reproductive senescence in human females is classified as ovarian follicle depletion [33]. Due to these reasons and others, caution should be exercised in extrapolating the results from rodent studies to humans. Although estrogen secretion by the ovary promotes hypothalamic changes, the chronic administration of estrogen can damage neurons in the arcuate nucleus and medial basal hypothalamus. Therefore, it can be important to remember that the surgical removal of the ovaries can lead to neurological changes that may affect other organs during the aging process.

### 2.3. Ovariotoxicity and Ovariectomy on the Aging Cardiovascular System

The pharmacological acceleration of ovarian aging using 4-vinylcyclohexene diepoxide (VCD) (accelerated ovarian failure (AOF) [34] model) or ovariectomy (OVX) procedures are often performed due to the lack of natural menopause in rat research models because they are believed to better mimic the hormone milieu seen in aging human females. VCD has been shown to target the plasma membrane of the primordial and primary follicles through direct inhibition of autophosphorylation of the oocyte-associated receptor, Kit [35]. Kit acts as an antiapoptotic factor in primordial follicular survival [36, 37]. Although an effective model of chemical ovotoxicity limited research has been performed using female rat models [38–41] and to date no published data exist regarding VCD and rat cardiovascular aging. VCD in juvenile (1 month) and adult (3 months) Sprague-Dawley rats depleted follicles but had no effect on the duration or onset of persistent estrus. However, FSH levels were significantly high in VCD treated animals with no change in cyclicity or serum levels of 17*β*-estradiol [38]. The absence of a human based clinical menopausal hormone profile in the AOF model brings into question the ability to apply data derived from this model for translational relevant treatment of cardiovascular human based menopausal pathologies.

An ovariectomy is the removal of the ovaries which induces a surgical menopause characterized by the cessation of estrogen and progesterone, as well as reduced production of testosterone. Surgical menopause leads to more severe and sudden symptoms compared to that observed during the natural human menopause where ovaries produce lower levels of hormones over time. Like that seen in menopausal human females, ovariectomy in rats also increases cardiovascular risk [42, 43].

Studies have found that the majority of human females who undergo natural menopause exhibit different age-associated cardiovascular alterations than those who underwent ovariectomy-induced menopause [44]. Elective bilateral removal of the ovaries at a young age is associated with an increased risk for cardiovascular disease and premature death [43]. In addition to declines in well-being and sexual function, it is thought that elective oophorectomies are also associated with elevated risk of cognitive impairment, dementia, and Parkinsonism [43]. It is not known whether the increased risk to cardiovascular disease is due to alterations in hormones or the hypothalamic-pituitary axis [43]. As expected, bilateral oophorectomy is associated with different hormonal alterations including changes in estrogen production and reduced levels of progesterone and testosterone, as well as increases in gonadotropins (LH and follicle stimulating hormone (FSH)), compared to those that occur in human females who experience natural menopause [45, 46].

In rats, ovariectomy causes alterations to heart structure and function which include increases in cardiac interstitial space, cardiac fibrosis, heart weight, and left ventricular weight [47]. Ovariectomy in rats has been shown to reduce cardiac contractility [48]. Ovariectomized rats appear to exhibit increased evidence of oxidative stress and cardiac apoptosis [47–51]. In rats, cytokine expression (tumor necrosis factor-alpha (TNF-*α*) and interleukin-1 beta (IL-1*β*)) and angiotensin converting enzyme (ACE) and angiotensin II type 1 receptor gene expression also appear to be increased following ovariectomy [48, 50]. When estradiol treatment was given to ovariectomized rats, it prevented the reduction of cardiac contractility as well as the increase in apoptosis and cytokine expression [48, 51]. Research in other rats, namely, aging female Dahl salt-sensitive rats, has shown that female rats are more likely to develop hypertension after ovariectomy [52]. The researchers postulated that estrogen protects against the increased activity of the renin angiotensin system. However, in human females, research has shown that postmenopausal hypertension occurs approximately five to 10 years after menopause, not immediately after starting menopause. Therefore, other age-related causes must occur independently of estrogen loss that promotes CVD in postmenopausal human females, as is shown by the fact that estrogen replacement therapy is not cardioprotective after menopause [53, 54]. Interestingly, both natural and surgically induced reproductive senescence rats exhibit increased FSH as well as decreased levels of estradiol and inhibin (A/B) [55]. Surgically induced reproductive senescence in rats has also been shown to alter dopamine receptor affinity in the heart [55].

Alterations in cardiac structure and function are not the only alterations found after ovariectomy. Compared to aged controls, human females that underwent prophylactic bilateral salpingo-oophorectomy exhibit increased total and low-density lipoprotein cholesterol levels [56]. In light of the fact that within human females natural menopause and ovariectomy results altered hormonal profiles and cardiovascular risk, the data suggest that age-associated cardiovascular disease in human females may be associated with both time dependent hormone deprivation and age-associated changes in structure, function, and protein signaling. Of note is the fact that most studies have used ovariectomized female rats at young ages (6–12 weeks old) to examine the effects of hormone deprivation on the cardiovascular system which can lead to unreliable results given that the cardiovascular system has not yet “aged” [57].

### 2.4. Cardiac Structure and Function in the Aging Female Rat

The increased risk of CVD in human females may be due to differences in the type or magnitude of age-associated alterations in cardiac structure and function. Olivetti and colleagues have demonstrated a small decrease in human heart weight with aging in males but not females [58]. Additionally, no significant change was noted with myocardium, left ventricular, or right ventricular free wall weight in the aging human female heart with no underlying cardiovascular pathology nor were age associated changes detected in proportion, shapes, size, or number of mononucleated and binucleated cardiac myocytes [58]. Given this information, it appears that natural aging in human females is not associated with cardiac remodeling during or after menopause in the absence of preexisting pathologies or loading abnormalities [58]. Similar conclusions were reached from echocardiographic studies [59]. Although a limited number of studies have looked at how cardiac structure and function change with age in female rats, very few have directly investigated how sex may affect cardiac structure and function. Go demonstrated that cardiovascular aging in male and female rats demonstrated significant gender differences in LV size and function [60].

Boluyt and colleagues investigated how aging may affect cardiac structure and function in female F344 rats using echocardiography [61]. Aging female rat cardiac structural changes included a dilatation of the left ventricle between 13 and 22 months of age. This dilatation was characterized by increases in posterior and septal wall thickness during diastole at 22 and 30 months of age [61]. Aging in the female F344 was also associated with increases in collagen content and collagen cross-linking [62]. In addition, Boluyt and colleagues demonstrated mild systolic dysfunction (decline in left ventricle ejection fraction, fractional shortening, and velocity of circumferential fiber shortening) in female 22-month-old animals when compared to young adults and that these changes preceded the development of mild diastolic dysfunction [61, 63]. These authors suggested that this modest decline in systolic function was due, at least in part, to a shift in the amount of alpha and beta myosin heavy chains [61, 62], similar to what is seen in the human failing heart [64]. Additional work, perhaps using other rat models, is needed in order to truly distinguish if these alterations are due to increasing age or if they are simply specific to the F344 strain.

Fannin and colleagues found that increased age in the female F344xBN was associated with increases in oxidative stress and damage [65]. Age-related changes in cardiac structure were also found, such as increase in heart to body weight ratio, cardiomyocyte cross-sectional area, posterior wall thickening, and left ventricle chamber dilatation. Further, increased age was also associated with diastolic dysfunction, alterations in heart rhythm intervals, and alternations in connexin 43 expression [65]. The presence of age-associated cardiac structural changes in the F344 and F344xBN animal models calls into question the use of these strains for translational data, in light of the absence of age-associated change in human females reported by Olivetti et al. [58]. Additionally, the menstrual cycle induced hormonal changes have been shown to cause cardiac functional fluctuations [66]. This fact could indicate that the time at which functional measurements are taken may impact the results and must be a consideration in comparing all female derived cardiac functional data.

### 2.5. Cardiac Arrhythmias with Aging

Aging is considered a risk factor for ventricular arrhythmia [67, 68]. In addition, aging is also associated with an increase in the percentage of incidence of atrial fibrillation [60, 69]. In male rats, age-associated increases in cardiac fibrosis as well as the changes in gap junction morphology have been hypothesized to cause changes in cardiac conduction in addition to the incidence of arrhythmias and death [68, 70]. As humans age, there is an increase in the EKG abnormalities which are associated with increased mortality [71]. Men tend to experience more atrial fibrillation, early repolarizations, Brugada syndromes, and sudden cardiac death than women, while women are more likely to be at risk to develop long QT syndrome-based arrhythmias as well as bradycardia-induced torsades de pointes [72]. Although sex related differences in cardiac electrocardiogram parameters have been observed, electrocardiogram abnormalities seem to vary between studies. In men, there is an increase in QRS duration and sinus cycle length while women tended to a have a higher maximum heart rate [73, 74].

Electrocardiogram analysis in elderly men and women has demonstrated large or intermediate Q waves, left axis deviation, negative T-waves, and complete right bundle branch block as well as atrial fibrillation/flutter [75]. In aging men, the QRS complex has been found to be narrowed, while aged men and women have been shown to undergo a leftward shift of the QRS axis [75]. Another work has suggested that puberty in men is associated with a shortened QT interval which is not present in women [76] and that aging in men demonstrates an increase in PR intervals and a prolongation of the QT interval [74]. Conversely, another research found that aging did not appear to alter EKG tracings in either men or women [74].

Similar alterations in electrocardiogram parameters have also been found in aging rats. In male Wistar rats, the PR and corrected QT intervals were found to increase with age [70]. Alterations in aging electrocardiogram parameters have been shown to be rat strain dependent and could only be detected subsequently to heart failure as in male SHHF/Mcc-cp rat strain at 19 months of age [77]. Gonadectomy appears to decrease the number of arrhythmias in male but not female rats [78]. Though not fully understood, these differences in gender associated gonadectomy induced arrhythmia frequency have been attributed to higher heart rates in male animals and longer QT intervals, as well as gender differences in ion channel expression [79]. Very few studies have investigated electrocardiogram parameters in the aging female rat. Fannin and colleagues found that the arrhythmias were not significantly higher in older female F344xBN rats when compared with younger rats; however, the study did find that valvular dysfunction was increased in the older rats when compared to the younger ones [65].

The exact mechanisms of the gender associated reduction of arrhythmias in humans and the role estrogen may play are not fully understood but two potential indirect mechanisms have been proposed. One potential indirect mechanism may be related to the ability of estradiol to reduce the incidence of cardiac ischemia [80]. Other works have suggested that high levels of estradiol in female and male rats may directly reduce arrhythmias, possibly by causing slowing of the inward calcium current [80, 81]. Although multiple studies have investigated EKG changes in aging rats, these parameters have not been determined in the NIA approved aging F344xBN rat model. However, according to the ICH-S7B, the use of rats for the safety testing of drugs is not considered appropriate due to differences in the ionic mechanisms of repolarization [82]. Arrhythmia mechanisms [83], resting heart rate, Ca^+^ homeostasis [84], and action potential configuration [85] are all important differences that limit the translational relevance of rat generated cardiovascular data for human application.

### 2.6. Aging Related Changes in Cardiac Oxidative Stress and ROS-Related Signaling

Aging in the male rat heart is associated with the accumulation of oxidative damage to lipids and proteins as well as decline in mitochondrial enzymes [86]. In addition, an increasing number of mutations in mitochondrial DNA (mtDNA) has been gradually observed during aging [87]. The level of 8-OHDG mtDNA adducts and deletions increase exponentially with age [87]. In human muscle, liver, and brain tissue, complex IV and mitochondrial oxidative phosphorylation enzyme activities decline with age. This decline in function is correlated with the accumulation of mtDNA mutations, including deletions, and base substitutions [87]. Similarly, aged male rat hearts exhibit an increase in superoxide, 4-hydroxynonenal (4-HNE), and nitrosative stress levels which appear to be highly correlated to increases in left ventricular thickness [88]. The exact role that increased levels of oxidative stress may play in the development and progression of age-associated cardiovascular disease remains to be determined.

Compared to that observed in females, the hearts of aging male rats exhibited increases in protein carbonylation, advanced oxidation protein products, nitrotyrosine, nonprotein thiol, reduced glutathione, and iron levels [89]. Although aging is associated with increases in oxidative stress in both male and female rat hearts, female rat hearts exhibited lower mitochondrial hydrogen peroxide production, oxidative damage, and a greater mitochondria differentiation compared to that seen in male animals [90]. It is thought that higher mitochondrial differentiation is a metabolic adaptation to increase energy efficiency as it is associated with their lower mitochondrial free radical production and oxidative damage [90]. In addition to differences with sex, it is also likely that reactive oxygen species (ROS) levels vary with animal strain as female Wistar and F344 rats exhibit lower ROS production and indices of oxidative damage than that seen in female Sprague-Dawley rats which may help to explain their greater mean lifespan [91, 92].

The mitogen-activated protein kinase (MAPK) cascades are evolutionary conserved serine/threonine protein kinases that regulate several important cellular functions including proliferation, differentiation, development, cell cycle, and cell death [93]. The major MAPK signaling pathways are the extracellular signal-regulated protein kinase cascade (p44/42), c-Jun amino-terminal kinase/stress-activated protein kinase cascade (JNK/SAPK), and the p38 cascade. Stimuli that include stress or injury typically activate the JNK and p38 MAPK kinases, while the p44/42 MAPKs are stimulated by mitogenic and growth factors [94, 95]. MAPK signaling is involved in many of the age-associated physiological changes (hypertrophy, oxidative stress, and apoptosis) as well as other cardiac pathologies. Works investigating MAPK signaling in aging human hearts have demonstrated reduced p38 MAPK activity/signaling in heart failure and impaired activation of MAPK target genes following increase in oxidative stress [96–98].

It is well recognized that increases in cellular stress cause the upregulation of the heat shock protein (Hsp) [99]. Hsps range in size from 10 to 150 kDa and function to prevent protein aggregation and assist in ensuring proper protein folding, as well as helping to mediate the translocation of damaged proteins [100]. Hsps are also involved in cardiac hypertrophy, in response to vascular wall injury, ischemic preconditioning, and aging [101]. During aging, there is an accumulation of damaged or misfolded proteins which may cause a burden on maintaining proteostasis [102–104]. An important function of heat shock proteins is to protect against age-related protein misfolding [103–105]. In aged male F344 rats (24 months), it appears that aging decreases Hsp70 upregulation following chronic exercise and heat stress [106–108].

**Figure 1:**
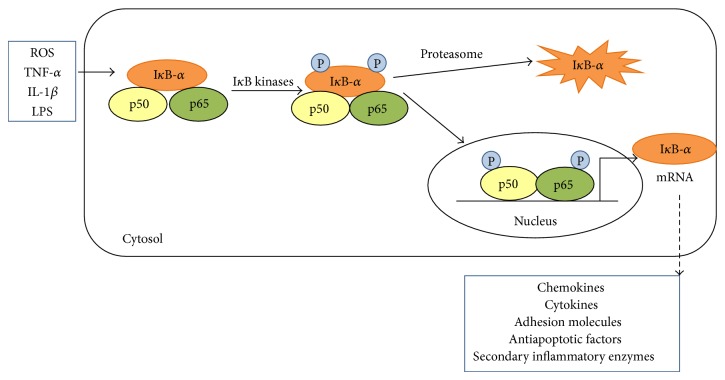
The NF-κB signaling pathway (see text for details).

Similar to the MAPK proteins, the nuclear factor-*κ*B (NF-*κ*B) pathway is thought to play a role in cardiac remodeling, apoptosis, acute ischemia and reperfusion, and unstable angina, as well as heart failure in both humans and rodents [109–112]. The NF-*κ*B family consists of RelA (p65), c-Rel (Rel), RelB, NF-*κ*B1 (p50), and NF-*κ*B 2 (p52).  Figure 1 presents the NF-*κ*B signaling pathway. In the cytoplasm, the p50 and p65 are found as an inactive heterodimer due to the binding of the I*κ*B kinase inhibitory proteins (IKK*α* and IKK*β*). Cellular stimuli known to activate the NF-*κ*B pathway include ROS, TNF-*α*, IL-1*β*, and bacterial lipopolysaccharides [113]. It has been shown that the I*κ*Bs must first become phosphorylated and degraded before p50/p65 activation can occur [113]. The p50/p65 when phosphorylated (activated) then translocates into the nucleus to induce the transcription of chemokines (IL-8, MCP-1), cytokines (TNF-*α*, IL-1, IL-2, IL-6), adhesion molecules (ICAM-1, VCAM-1, E-selectin), acute phase proteins, antimicrobial peptides, secondary inflammatory enzymes (COX-2, iNOS, PLA2, MnSOD), and antiapoptotic factors [114]. Upon termination of the NF-*κ*B stimuli, p50/p65 binds to new I*κ*Bs and the complex is translocated back to the cytoplasm. It is thought that the MAPK pathway works in concert with the NF-*κ*B pathway to increase transcription of inflammatory genes [114]. Meldrum et al. extensively review the role of sex hormones in intracellular signaling mechanisms within the myocardial inflammation [66].

### 2.7. Activation of ROS-Related Signaling in the Female Aging Heart

Although the effects of estrogen on MAPK signaling have been studied in several pathological processes, for example, breast cancer, migraines, and polycystic ovarian syndrome, little is known regarding how this molecule affects MAPK signaling in the heart. It is thought that differences in MAPK activation between male and females may be regulated, at least in part, by the influence of estrogen [66, 115, 116]. Estrogen has been shown to stimulate the activation of p44/42 MAPK and JNK MAPK in different model systems including cardiomyocytes, mammary cancer cells, pituitary tumor cells, tissue slices of the hippocampus, and endometriotic stromal cells [117–119]. In males as well as ovariectomized females MAPK signaling is decreased after acute ischemia [115, 116]. In a model of cardiac pressure overload, estradiol treatment appeared to exhibit antihypertrophic effects by increasing the expression of atrial natriuretic peptide (ANP) and inhibiting p38-MAPK activation [120].

In addition to the regulation of MAPK proteins, there is evidence to suggest that gender also has an effect on the Hsp response. In hearts that were not exposed to increased cellular stress, male hearts have half as much Hsp72 expression compared to that observed in the female heart. Conversely, ovariectomy was found to reduce Hsp72 levels in female hearts [121]. Estradiol treatment has also been found to increase Hsp27, Hsp70, Hsp72, and Hsp90 expression in the heart [122–124]. To our knowledge, no studies have looked at Hsp27, Hsp70, and Hsp90 in the aging female heart.

Aging female and male hearts have shown increased apoptosis and inflammation, as well as age-associated gender differences in NF-*κ*B signaling [125]. To our knowledge, only a few studies have investigated the changes in NF-*κ*B expression and activity in the aging female rat heart. However, it has been reported that estradiol can decrease NF-*κ*B binding activity [66].

### 2.8. Aging Heart Apoptosis

Apoptosis or programmed cell death is a highly conserved and regulated cell response to inhibit the abnormal proliferation of cells [126]. Cardiomyocytes are not capable of self-regeneration and are terminally differentiated but can undergo apoptosis, necrosis, or autophagy when unduly stressed [127]. Apoptosis is increased in many cardiovascular diseases such as dilated and ischemic cardiomyopathy, hypertrophic heart disease, and arrhythmias [126, 128, 129]. In rats, increased age has been shown to cause apoptotic susceptibility in the heart following oxidative injury [130, 131]. Aging in the male F344xBN heart is characterized by increases in the amount of cytochrome* C*, AIF, Bax, rate of permeability transition pore opening, and fragmented DNA [132]. The age-associated increase in apoptosis appears to be due not only to the activation of proapoptotic molecules but also to decreases in antiapoptotic NF-*κ*B, Bcl-xL, and Grx1 signaling [130]. Another work has demonstrated that the aging male F344xBN rat heart is characterized by increases in TdT-mediated dUTP nick end labeling- (TUNEL-) positive nuclei, caspase-3 activation, caspase-dependent cleavage of alpha-fodrin, and diminished phosphorylation of protein kinase B/Akt (Thr308) [133]. The increase of apoptosis in the aging male F344xBN was highly correlated to age-associated increases in oxidative-nitrosative stress. Though not demonstrating cause and effect, these results suggest that increased cellular ROS and cardiomyocyte apoptosis may play a role in age-related cardiac remodeling.

The incidence of cells undergoing apoptosis in the heart has been shown to differ with sex in humans and rats. In humans, a higher number of TUNEL positive myocytes are seen in young males compared to females [58, 134]. In humans, aging appears to increase cardiac apoptosis in male but not female [58]; however, there was no gender difference in apoptosis in male and female F344 rat [92, 135]. These results suggest differences in apoptosis regulation between species.

### 2.9. Effects of Aging on Aorta Structure and Function

Cardiovascular disease affects not only the heart but the vasculature of the cardiovascular system. Structural changes found in the aging vasculature include an enlarged lumen, media-intimal thickening, irregularly shaped endothelial cells, migration and proliferation of vascular smooth muscle cells, increased deposition of extracellular matrix, increased expression of adhesion molecules, and alterations in the expression of metalloproteinases as well as cytokines [4, 136–138]. Functional changes with age in the vasculature consist of impaired distensibility, increased stiffness, increased endothelial permeability, attenuation of *β* 2-adrenoceptor vasodilator response to agonists, and diminished response to adrenergic receptor stimulation [4, 139]. Like humans, rats also appear to exhibit several age-associated changes in vascular structure and function including changes in intimal thickening, elevations in inflammation-associated molecule expression, and increased evidence of oxidative stress [4, 140].

Similar to that seen in other parts of the cardiovascular system, differences in aortic structure and function between genders have also been observed in the aging human and animals. In males, aortic wall and intimal-medial thickness are greater during aging than that observed in women; however, stiffness was not different between men and women [141]. Similarly, there were no differences in distensibility in the aging aorta between men and women [142]. Although the human aging aorta did not show increases in aortic stiffness, it has been documented that the increased vascular stiffness with aging is more prominent in male than female animal models [143, 144]. This may be due in part, to the lower total peripheral resistance and higher cardiac output seen in women compared to men of similar arterial pressure and age [58].

Abnormal proliferation and migration of vascular smooth muscle cells (VSMCs) play important roles in the pathophysiology of atherosclerotic diseases [145]. Previous studies have shown that VSMCs isolated from old animals replicate more actively than those obtained from young animals [145, 146]. Similarly, aging has been shown to be associated with an increased proliferative response of VSMCs after balloon angioplasty [147].

### 2.10. Effects of Aging on Aortic Endothelial Cell Function

Endothelial dysfunction is considered to be a common and early feature of vascular disease and impaired endothelium-dependent relaxation has been demonstrated in several rat models of hypertension, experimental diabetes, atherosclerosis, and high salt diet, as well as aging [148]. The factors regulating endothelial dysfunction are likely complex and may vary between models and with aging. Nitric oxide (NO) is a vasodilator that is produced by endothelial nitric oxide synthase (eNOS) that plays a crucial role in blood pressure regulation. NO production is stimulated by shear stress, cyclic strain, acetylcholine, vascular endothelial growth factor, bradykinin, estrogen, sphingosine-1 phosphate (S-1P), hydrogen peroxide, and angiotensin II [149]. It is thought that eNOS activity is regulated by eNOS phosphorylation at Serine 1177, Serine 635, and Serine 617 by phosphatidylinositol 3-kinase (PI3K)/Akt, adenosine monophosphate-activated protein kinase (AMPK), and cyclic AMP-dependent protein kinase signaling [149, 150]. Given its important role in regulating vascular function, it is not surprising that abnormalities in vascular NO production are thought to contribute to the pathogenesis of atherosclerosis and hypertension [151, 152]. Advanced age has been found to be associated with impaired endothelial NO synthesis and endothelial dysfunction [153, 154]. The mechanisms responsible [155] for age-associated alterations in NO synthesis are not fully known but may include changes in activity or expression of eNOS, increased breakdown of NO due to oxidative stress/oxidative injury, changes in antioxidant defense systems, and decreased availability of eNOS substrate, L-arginine [155], although the activity of eNOS is generally thought to be diminished with aging [156] in the aorta. In addition to changes in eNOS activity, decreased NO availability during aging can also be caused by oxidative stress [154] and alterations in eNOS structure [155, 156]. The regulation of eNOS function during aging may also differ with sex. Although estrogen (17-*β* estradiol) has been shown to activate eNOS [157], estrogen studies in rats have shown no effect [158] or increased eNOS expression following estrogen treatment [159]. Conversely, in humans, estrogen replacement appears to be largely ineffective as a means of decreasing CVD risk [159, 160].

### 2.11. Sex Related Differences in Aortic Vascular Smooth Muscle Cells with Aging

In women, the incidence of vascular dysfunction is thought to be related, at least in part, to the cessation of ovarian hormone production [161]. In vascular smooth muscle cells from young female Wistar Kyoto rats, estrogen inhibited VSMC proliferation following stimulation with fetal calf serum [162]. This effect was thought to be mediated by E2 receptors [162]. Similar decreases in VSMC proliferation with estrogen treatment following cell stimulation by growth factors or mechanical stress have also been noted [161, 163]. Like estrogen, progesterone is also thought to inhibit the proliferation of aortic VSMC, most likely via its ability to inhibit DNA synthesis [164]. Nonetheless, it should be noted that the effects of estrogen in vivo are likely more complex than those seen in cell culture. For example, in aortas from diabetic female rats, estrogen failed to reverse the impaired basal release of nitric oxide and the abnormal relaxation to histamine [165].

### 2.12. Aging Aorta Signaling

The MAPK signaling pathways function to regulate many processes in the aorta including VSMC proliferation, contraction, migration, differentiation, and cell survival [166–168]. Aging has also been shown to increase the activation (phosphorylation) of p44/42 and JNK MAPKs in the aorta [169] while another work has shown that aging affects the ability of the aged aorta to activate p38 and JNK MAPK signaling following mechanical loading [170]. In aged animals the activation of p44/42 MAPK in vascular smooth muscle was increased compared to young animals [145, 171].

Similar to that observed for the heart, age-associated changes in the expression/activity of NF-*κ*B were found in the vascular smooth muscle cells of the aorta. Aging has been found to increase the sensitivity of NF-*κ*B to glucose in aortic VSCM cells [172]. Another research has shown that VSCM proliferation and NF-*κ*B activation stimulated by interleukin-1*β* were increased more in aged female rats than young female rats [173]. In premenopausal women, receiving hormone treatment appeared to reduce NF-*κ*B activation [174] suggesting that estradiol reduces NF-*κ*B activation. How estrogen may inhibit NF-*κ*B activity is currently unclear but may be related to increased I*κ*B levels or decreased levels of circulating TNF-*α* [174].

## 3. Conclusion

In summary, the loss of ovarian function during aging may influence cardiovascular structure and function. However, the fact that human females do not display cardiac hypertrophy or myocyte loss in the absence of loading abnormalities with aging may suggest the presence of estrogen prior to menopause acts to attenuate the cardiac response to preexisting underlying pathologies. Due to the complexities of the aging process, more studies are needed to distinguish whether observed differences are due to aging alone, hormone deprivation, or a combination of both. In order to overcome the difficulties found in aging human research (ethics, cost, and long lifespan), rat models have been used to provide great insight in investigating age-related changes in cardiovascular structure and function. However, additional research examining how aging may affect cardiac structure and function in rats is needed to determine whether the alterations seen in rats mimic the changes seen in humans. Because of the systemic nature of reproductive senescence, the use of surgical practices to induce “pseudomenopause” or ovariotoxicity may be incomplete in the overall approach to female aging and cardiovascular changes. The global changes in the body with age may play a cumulative role in combination with the hormonal changes seen in reproductive senescence. Aging-associated increases in oxidative stress can lead to alterations in the cardiac cell signaling which may lead to deleterious changes in the heart. Because cardiac structure, function, signaling, and hormonal response appear to differ between male and female rats and humans with aging, care must be taken in evaluating current literature with regard to specific finding and gender. Studies investigating the details of cardiac signaling with gender and aging are likely to lead to a better understanding of the mechanisms responsible for cardiovascular diseases in both male and females. These findings may prove critical in the application of current prophylactic practices and pharmaceutical interventions in patient outcomes.

## Figures and Tables

**Table 1:** Stages of ovarian aging in a female rat as described by Lu et al. (1979) [24].

Age (months)	Stages of ovarian aging in female rats
5 months	Reproductive maturity
	(i) Estrous cycle (4-5 days)
	(ii) Elevated and constant levels of estradiol
	(iii) Low levels of progesterone
	(iv) Lack of hormone surges and ovulation

6–8 months	Ovarian decline
	(i) Decreased levels of estradiol and progesterone
	(ii) Little or no follicles

10–12 months	Irregularity of estrous cycles

19 months	Constant estrous cycle
	(i) Low to medium levels of serum estradiol, estrone, testosterone, androstenedione, and progesterone
	(ii) Very low levels of 20α-hydroxyprogesterone
	(iii) No preovulatory release of gonadotropin and prolactin
	(iv) Increased levels of FSH

24 months	Prolactin
	(i) Increased levels of prolactin (abolished by ovariectomy)
	(ii) Retained ability to develop follicles and corpora
	(iii) Retained ability to secrete steroid hormones
